# Genetic Interplay Between Attention-Deficit/Hyperactivity Disorder and Pain Suggests Neurodevelopmental Pathways and Comorbidity Risk

**DOI:** 10.1016/j.bpsgos.2025.100517

**Published:** 2025-04-25

**Authors:** Nicolas P. Ciochetti, Victor F. de Oliveira, Iago Junger-Santos, Cibele E. Bandeira, Maria E. Tavares, Eduardo S. Vitola, Luis A. Rohde, Gustavo Melo de Andrade, Bruna S. da Silva, Eugenio H. Grevet, Claiton H. Dotto Bau, Diego L. Rovaris

**Affiliations:** a Laboratory of Physiological Genomics of Mental Health, Instituto de Ciências Biomédicas, Universidade de São Paulo, São Paulo, Brazil; b ADHD Outpatient Program & Developmental Psychiatry Program, Hospital de Clínicas de Porto Alegre, Universidade Federal do Rio Grande do Sul, Porto Alegre, Brazil; c Departamento de Psiquiatria, Faculdade de Medicina, Programa de Pós-Graduação em Psiquiatria e Ciências do Comportamento, Universidade Federal do Rio Grande do Sul, Porto Alegre, Brazil; d Medical Council, UniEduK and Unifaj, Indaiatuba, Brazil; e Center for Research and Innovation in Mental Health, National Institute of Developmental Psychiatry, São Paulo, Brazil; f Center for Teaching and Research in Brain Aging (NUDEC), Federal University of São Paulo, São Paulo, Brazil; g Department of Basic Health Sciences, Universidade Federal de Ciências da Saúde de Porto Alegre, Porto Alegre, Brazil; h Departamento de Genética, Instituto de Biociências, Programa de Pós-Graduação em Genética e Biologia Molecular, Universidade Federal do Rio Grande do Sul, Porto Alegre, Brazil

**Keywords:** Attention-deficit/hyperactivity disorder, Genome-wide association study, Migraine, Pain, Pleiotropy, Polygenic risk score

## Abstract

**Background:**

In this study, we investigated the genetic connections between attention-deficit/hyperactivity disorder (ADHD), migraine (MGN), and multisite chronic pain (MCP). The goal was to identify specific shared biological mechanisms that contribute to the overlap between ADHD and these pain-related conditions.

**Methods:**

We utilized various post–genome-wide association study analyses on summary data from samples ranging between 225,534 and 766,345 individuals. In an independent sample of patients with ADHD and control participants (665 cases and 995 controls), we evaluated MGN and MCP polygenic risk scores (PRSs) in relation to comorbid profiles, symptom severity, and neuroimaging brain scores.

**Results:**

The findings show a strong biological overlap between ADHD and MCP, with a less pronounced relationship with MGN. Key regions and genes associated with ADHD and MCP were enriched in neurodevelopmental pathways, including those involved in neuron projection morphogenesis and nervous system development. Drug-set enrichment analysis identified that some of these pathways are potentially influenced by paracetamol, a drug that has been implicated as a class I environmental risk factor for ADHD when exposure occurs prenatally. Causal inference analysis using a 5-fold larger ADHD summary dataset demonstrated stronger effects of MCP on ADHD than the reverse. In the independent sample, higher MCP PRSs were linked to structural brain features, increased comorbidity with substance use and bipolar disorder, and heightened severity of ADHD symptoms.

**Conclusions:**

These findings underscore the significant genetic relationship between ADHD and MCP, suggesting that shared genetic factors may influence brain development and contribute to diverse clinical outcomes in ADHD.

## Plain Language Summary

In this study, we investigated genetic links between attention-deficit/hyperactivity disorder (ADHD), migraines, and multisite chronic pain (MCP). The results reveal a strong genetic connection between ADHD and MCP, emphasizing shared biological pathways involved in brain development. By analyzing genetic data, researchers found that higher genetic risk for MCP was associated with changes in brain structure, greater ADHD symptom severity, and increased risk of other mental health conditions. These findings suggest that shared genetic factors may play a key role in shaping brain development and influencing diverse clinical outcomes in ADHD.

The etiology of attention-deficit/hyperactivity disorder (ADHD) is complex, involving a dynamic interplay between innate predispositions and environmental influences, with genetics accounting for ≈80% of the phenotypic variance (1). Notably, ADHD rarely exists in isolation, often co-occurring with a spectrum of neuropsychiatric and somatic conditions, including depression, sleep disorders, and metabolic and inflammatory illnesses (1, 2, 3). Clinical and epidemiological studies have revealed a higher-than-expected comorbidity between ADHD and migraine (MGN) (4,5). MGN is significantly more prevalent among individuals with ADHD than control individuals, with both genders showing an increased risk of developing this comorbid condition (5). Additionally, emerging research suggests a link between ADHD and multisite chronic pain (MCP) (6,7). Conversely, individuals with ADHD report a higher prevalence of pain, a pattern that has been observed consistently across various countries (8). It has been proposed that in adults, persistent pain influences attention, cognitive abilities, and the control of emotional responses. Similarly, motor regulation problems may serve as a link, particularly connecting hyperactivity/impulsivity, inattention, and the experience of pain (7,9).

MGN symptoms are wide ranging but are primarily characterized by throbbing acute unilateral headaches and pain and extreme sensitivity to light, sounds, movement, and even touch, often accompanied by nausea, stiff neck and back, fatigue, and irritability, among others (10). It can be a chronic condition, consisting of episodic attacks sometimes triggered by stimuli such as sleep deprivation or oversleeping, stress, hunger, or even certain foods. However, due to the complexity of this condition, the precise mechanisms fundamental to the initiation, maintenance, and termination of migraine attacks and pain remain unclear (10,11). Similarly, MCP consists of a broad phenotype that involves pain reported in more than 1 body site for at least 3 months. It is often comorbid with various neurological and non-neurological conditions, such as musculoskeletal disorders, cancers, immune system diseases, and other forms of pain (12,13). Both the immune and nervous system are implicated in the etiology of MCP (14).

At the genome-wide level, there have been broader investigations into the genetic relationships between MGN, MCP, and mental disorders (6,15, 16, 17). Johnston *et al.* demonstrated that the global genetic correlation between ADHD and MCP is twice as strong as that between ADHD and MGN (*r*_g_ = 0.56 vs. *r*_g_ = 0.26, respectively) (6). Moreover, Mendelian randomization (MR) analysis has identified MCP as a risk factor for ADHD and vice versa (15). Recently, a comprehensive multiancestry genome-wide association study (GWAS) of pain reinforced the significant positive genetic correlations between ADHD and pain intensity (16).

Although existing research shows strong genetic correlations between ADHD and pain-related phenotypes, no study has thoroughly explored these relationships. This gap warrants further investigation, because a deeper understanding could significantly enhance our knowledge of the underlying biology of ADHD and its comorbidities. In this study, we conducted a comprehensive exploration of the biological relationships between ADHD, MGN, and MCP using large-scale GWAS data. We also examined the influence of MGN and MCP polygenic risk scores (PRSs) on various aspects of ADHD, including its clinical profile, severity, and structural brain aspects using a thoroughly independent and well-characterized ADHD sample.

## Methods and Materials

### GWAS Summary Statistics

We obtained the latest available GWAS summary statistics. The ADHD dataset includes 38,691 individuals with ADHD and 186,843 control participants without ADHD (18). For MGN, the dataset comprises 48,975 cases and 540,381 controls (19). Data for MCP, totaling 412,985 individuals, was accessed through the PanUKBiobank (https://pan.ukbb.broadinstitute.org/).

Considering the well-established genetic correlations between major depressive disorder (MDD) and ADHD, MGN, and MCP, as well as between educational attainment (EA) and ADHD, MGN, and pain (6,13,16,17,20), we included these phenotypes as potential confounders to mitigate results that might be influenced by factors strongly associated with ADHD. To account for confounding, we performed a sensitivity analysis by refining local genetic correlations through the incorporation of MDD and EA as third traits in a partial correlation analysis. For MDD, we relied on summary statistics from a large-scale study that incorporated data from 264,984 cases and 581,929 controls (21). Regarding EA, we utilized the most up-to-date GWAS summary statistics with 766,345 individuals (22). For a detailed description of the base samples used, together with quality control (QC) procedures, see the Supplement.

### Quantification of Total Polygenic Overlap

In this study, we present updated findings based on a more extensive ADHD GWAS meta-analysis that was conducted with a dataset 5 times larger than previous research (6,17,23). The genetic correlations were computed utilizing cross-trait linkage disequilibrium (LD) score regression (24,25). Polygenic overlap was measured through univariate and bivariate causal mixture (MiXeR version 1.3) (26) as described in detail in the Supplement.

### Local Genetic Correlations

We used LAVA (27) to pinpoint genomic regions that influence global genetic correlations, focusing on ADHD versus MGN and ADHD versus MCP. LAVA partitions the genome into 2495 semi-independent LD loci with a minimum size of 1000 single nucleotide polymorphisms (SNPs) per locus. Significant loci are those with a *p* value < .05 after Bonferroni correction. Partial genetic correlations were also assessed to account for MDD and EA confounding influences (see the Supplement for detailed methods).

### Conjunctional False Discovery Rate

We applied the conjunctional false discovery rate (conjFDR) to identify additional variants associated with the investigated pair of traits (28,29) (Supplement). Different from LAVA, which identifies specific regions driving global correlations, conjFDR pinpoints specific shared variants, thereby providing complementary insights. Using conjFDR and LAVA as a combined approach enhances the ability to identify genes and functional pathways potentially relevant to the phenotypes. To evaluate the concordance between these methods, we compared the SNPs identified by conjFDR with the regions defined by LAVA and examined whether any SNPs highlighted by conjFDR were located within LAVA-delimited regions.

### Enrichment Analysis

Gene-set analysis for each LAVA locus was conducted with Enrichr (30), utilizing Reactome 2022 pathways, Gene Ontology biological process, cellular component, and molecular function. SNPs significant in conjFDR were annotated with ANNOVAR (31), and intergenic variants were mapped to the nearest genes before enrichment analysis.

#### Drug-Set Enrichment Analysis

The only environmental factor that has been shown to confer risk for ADHD with class I evidence is paracetamol use during pregnancy (32). However, maternal pain and/or ADHD may be relevant confounders in this association, which introduces uncertainty about causality (33). Thus, we examined whether any identified genes or pathways retrieved by LAVA and conjFDR intersect with the pharmacology of paracetamol (see the Supplement). To explore this intersection of known genes/pathways and paracetamol pharmacology, we used DGIdb 5.0 (34), BindingDB (35), and Psychoactive Drug Screening Program Ki databases (36), searching for both “paracetamol” and “acetaminophen.” Furthermore, drug-set enrichment analysis (DSEA) was used to investigate shared mechanisms and compare these pathways with results from LAVA and conjFDR analyses to identify commonalities with paracetamol’s effects.

### MR: Genetic Instrumental Variable Analysis

We conducted bidirectional 2-sample MR between ADHD and MGN and MCP using MR-Egger, weighted median, inverse variance-weighted (IVW), robust adjustment profile score, and contamination mixture (37, 38, 39). Sensitivity analyses included Cochran’s *Q* heterogeneity tests, MR-Egger regression intercepts, Steiger directionality tests, funnel plots, and leave-one-out analysis (40,41). To select the instrumental variables, we clumped the ADHD summary statistics selecting SNPs reaching a *p* < 5 × 10^−8^ with a 1000-kb window and an *r*^2^ = 0.01 (42). Then, we harmonized the exposure beta and standard error coefficients with those calculated for the SNPs associated with the outcomes (MGN and MCP) to perform the MR. Ambiguous and palindromic SNPs were excluded. We used the R packages *Two-Sample MR* (37), *Mendelian Randomization* (43), and *mr.raps* (38) to conduct the analyses. Results consistent across at least 4 methods were considered significant, with FDR applied for multiple testing correction. Details on the methods used are provided in the Supplement.

### Analysis in an Independent Single-Site Case-Control Sample

#### Sample Description

The sample consisted of 665 adult individuals with ADHD from the ADHD Outpatient Program at Hospital de Clínicas de Porto Alegre (HCPA) and 995 blood donor control participants from the same hospital. ADHD diagnosis followed the DSM-IV criteria from 2001 to 2012 (44) and DSM-5 criteria from 2013 onward (45,46). Lifetime diagnoses of ADHD and oppositional defiant disorder were conducted using the Portuguese version of the Kiddie Schedule for Affective Disorders and Schizophrenia adapted for adults (47). Other lifetime psychiatric comorbidities were assessed by trained psychiatrists using the Structured Clinical Interview for DSM-IV (SCID-IV) Axis I disorders from 2001 to 2012 (48), an adapted version of the SCID from 2012 to 2015, and the SCID-5 from 2015 onward (49). The severity of ADHD symptoms was obtained using the 18-item Adult ADHD Self-Report Scale (ASRS) (50). The control sample screened negative for ADHD, evaluated using the 6-item ASRS (51). All participants were fully briefed on study procedures and provided signed consent, approved by the HCPA Institutional Review Board (IRB) (IRB 0000921) in accordance with the Declaration of Helsinki and Brazilian regulations.

Blood samples were drawn from all participants, from whom DNA was extracted and genotyped following the steps detailed in the Supplement. Briefly, a portion of the sample was genotyped using the Infinium PsychArray BeadChip (Illumina), while the remainder was genotyped with the Infinium Global Screening Array BeadChip versions 1.0+MD and 3.0+MD (Illumina). The use of different chips resulted from genotyping in separate batches, and the QC procedures were adjusted accordingly.

#### Ancestry Inference

The ancestry distribution of cases and controls against 1000 Genomes populations was assessed using multidimensional scaling (MDS) (52). Thus, we used the values of MDS components 1 and 2 from the 1000 Genomes European reference groups to classify the European individuals in our sample, while the remaining individuals were classified as admixed.

#### PRS Calculation and Analysis

PRSs for MGN and MCP were calculated using the largest GWAS as the reference sample [(19) and PanUK Biobank]. We computed PRSs using the continuous shrinkage method (53). It considers the LD patterns of SNPs to implement continuous pruning of genetic variants, demonstrating robustness across diverse genetic architectures and suitability for multivariate modeling of local LD patterns (54).

#### Neuroimaging

Structural T1-weighted magnetic resonance imaging (MRI) data were acquired using a Siemens Magnetom Spectra 3T scanner for 121 ADHD cases and 82 controls. For detailed information on MRI data acquisition, preprocessing, and QC, refer to previous studies (55,56). Anatomical segmentation of T1-weighted 3-dimension magnetization-prepared rapid gradient-echo (MPRAGE) sequences was performed using FreeSurfer version 5.3 (57), following ENIGMA (Enhancing Neuro Imaging Genetics through Meta Analysis) Consortium protocols (https://enigma.ini.usc.edu/).

Brain scores (neuroimaging association scores [NASs]) for ADHD were calculated following the methodology published by Axelrud *et al.* (58). NASs were derived using Cohen’s *d* values from ENIGMA studies (59,60), considering *p* thresholds of .05, .5, and 1, together with an additional *p* threshold FDR score. The scores were computed in RStudio by regressing out covariates from the structural measures in our sample, transforming the residuals into *z* scores, and multiplying these *z* scores by the Cohen’s *d* values provided by ENIGMA. The resulting values were summed to generate modality-specific scores for cortical thickness, surface area, and subcortical volume. Brain scores with a *p* threshold of 1 were selected for further analysis because they demonstrated the highest reliability (55). A detailed description can be found in the Supplement.

#### Statistical Analysis

We investigated the correlations between PRSs for MGN and MCP and various outcomes in the ADHD sample. The outcomes included case-control status, comorbid profiles, and ADHD symptom severity. We performed logistic regression for categorical outcomes (e.g., case-control status) and linear regression for continuous outcomes (e.g., severity of ADHD). The models included sex, age, and the first 5 principal components as covariates. When the sample size was appropriate, we performed an additional sensitivity analysis including only Brazilians of European ancestry.

For analyses involving brain scores, we included interaction terms between each PRS and case-control status in the regression models to evaluate whether the genetic liability, as reflected by the PRS, had different effects in cases and controls. Specifically, we examined the interaction between the polygenic score for MCP and MGN and case-control status on brain scores, based on the premise that 1) brain structural variation is heritable and polygenic, and 2) ADHD brain scores reflect a neurodevelopmental dimension that persists into adulthood (58,61). These factors underscore the relevance of brain scores in exploring the relationship between genetic predispositions for pain traits and brain structure across diagnostic groups.

## Results

### Polygenic Overlap

**Figure 1:**
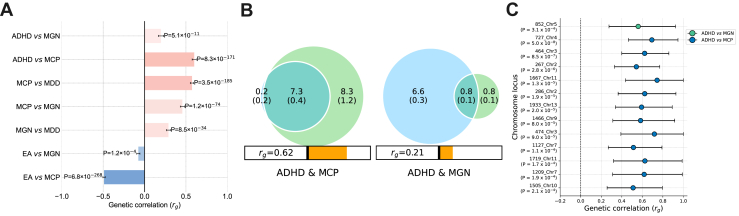
**(A)** Summary of global genetic correlations (*r*_g_s) among ADHD, MGN, MCP, MDD, and EA. All correlations were significant after Bonferroni correction (alpha = .05/6). **(B)** MiXeR Venn diagrams showing the variant overlap between ADHD and MGN and ADHD and MCP. Variants unique to ADHD are shown in blue, unique to MCP or MGN in light green, and shared variants in dark green. Numbers are reported in thousands with standard deviations in parentheses. The *r*_g_ values are shown below, with orange right-filling bars representing positive correlations (see Tables S1 and S2). **(C)** Summary of local *r*_g_s for ADHD vs. MGN (green) and ADHD vs. MCP (blue) from LAVA. All correlations were significant after Bonferroni correction (alpha set at .05/119 for ADHD vs. MGN and .05/240 for ADHD vs. MCP). The x-axis represents the correlation values, while the y-axis indicates the loci identified by LAVA along with their chromosomal locations. ADHD, attention-deficit/hyperactivity disorder; EA, educational attainment; MCP, multisite chronic pain; MDD, major depressive disorder; MGN, migraine.

First, we reexamined the genetic correlations among ADHD, MGN, and MCP using the latest GWAS summary statistics. Our findings are consistent with existing literature that has shown consistently positive correlations between these traits (Figure 1A). It is worth highlighting that the *r*_g_ between ADHD and MCP is notably higher (*r*_g_ = 0.6) than that between ADHD and MGN (*r*_g_ = 0.2). MiXeR analysis also showed considerable polygenic overlap between ADHD and MCP but not between ADHD and MGN (Figure 1B). From the 7500 variants estimated to influence ADHD that can explain 90% of the SNP-based heritability, 7300 were shared with MCP. On the other hand, only 800 variants were shared between this disorder and MGN (Figure 1B and Figure S1). Furthermore, there was a reported mean of 88% of concordance within shared variants among ADHD and MGN, as well as among ADHD and MCP, meaning that most variants did not differ in effect direction (Table S1).

The local correlation analysis showed specific loci relevant to the global correlations. Locus 852 presented a positive correlation between ADHD versus MGN (see Figure 1C and Table S2). As for ADHD versus MCP, LAVA yielded 12 loci (Figure 1C and Table S3). The full list of genes within each locus is provided in Table S4.

The enrichment analysis conducted for locus 852 (ADHD vs. MGN) identified 10 gene sets, including “stem cell development” and “neural crest cell differentiation.” However, only “siRNA binding” retained significance after correction for multiple comparisons (Table S5). The remaining significant loci retrieved by LAVA (ADHD vs. MCP) amounted to well over 300 significant terms (*p* value < .05), although only 82 of them had a *q* value < .05. Given the variety of genes, the associated biological pathways and functions varied as well. For example, genes at locus 286 participate in pathways related to “brain morphogenesis,” “neuron projection morphogenesis,” “brain development,” and several other neurodevelopmental gene sets (Table S5). Locus 1933 contains the *GPC5* and *GPC6* genes. The proteins encoded by these genes play a role in controlling cell division and growth regulation. Similarly, locus 1466 was associated with “negative regulation of cell cycle.” Furthermore, locus 1719 includes genes such as *NCAM1* and *DRD2*. It is enriched for “serotonin receptor activity” and “neurotransmitter receptor complex,” in addition to multiple other pathways related to synaptic transmission function (Table S5).

To determine whether the significant loci are sensitive to the inclusion of relevant third phenotypes, we performed a secondary analysis by refining these loci through partial correlation analyses incorporating MDD and EA. Because the only locus from ADHD versus MGN (locus 852) and 6 loci from ADHD versus MCP (267, 286, 464, 727, 1466, and 1719) (Table S6) presented local correlations with either MDD, EA, or both, we performed partial correlation analysis conditioning on MDD and EA in these loci. For locus 852, when conditioning on MDD, the correlation decreased, but the locus remained significant, which indicates an independent association between ADHD and MGN regardless of MDD (Table S7). The local *r*_g_ and *p* values increased and decreased, respectively, after accounting for EA. The correlation between ADHD and MGN was positive, while the correlation between ADHD and EA was negative. Partialling out the shared variance with EA may remove a negative, which could explain this finding (18). For locus 267 (ADHD vs. MCP), the local correlation lost its relevance when MDD was controlled for. Likewise, for loci 464, 727, 1466, and 1719, the *p* values lost their significance when conditioned on EA. These findings suggest that MDD and/or EA may more effectively explain the correlation between ADHD and MCP at these 5 loci.

**Figure 2:**
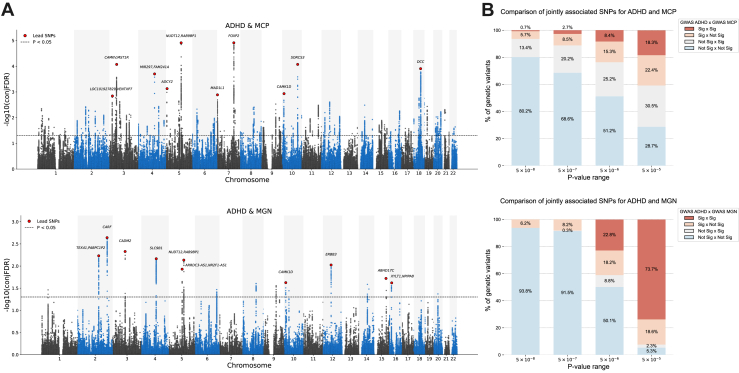
**(A)** ConjFDR Manhattan plots for ADHD and MCP (top) and ADHD and MGN (bottom). The x-axis displays each chromosome, and the y-axis shows the −log_10_ of the conjFDR *p* value for each SNP tested. The dashed line indicates the significance threshold (−log_10_[conjFDR < .05]). The top 10 lead SNPs from significant loci are highlighted in red and labeled with their nearest gene. **(B)** Comparison of jointly associated SNPs in the conjFDR analysis for ADHD and MCP (top) and ADHD and MGN (bottom) using different significance thresholds in the original GWAS. Novel variants are highlighted in light blue. The x-axis represents the different *p*-value thresholds, while the y-axis shows the percentage of SNPs tested in the conjFDR analysis that were previously associated with the investigated traits in their respective original GWAS. ADHD, attention-deficit/hyperactivity disorder; conjFDR, conjunctional false discovery rate; GWAS, genome-wide association study; MCP, multisite chronic pain; MGN, migraine; Sig, significant; SNP, single nucleotide polymorphism.

ConjFDR analysis also corroborated the extensively shared genetic background between ADHD and MCP. It revealed 191 loci jointly associated variants with the 2 traits as shown in Figure 2A, Figure S2, and Table S8. These SNPs were annotated to 226 genes within a 20-kb window (protein coding or not), 201 of which were not annotated as hits in the original GWASs on these phenotypes (see Table S8 for the full list of annotated genes). At the single-variant level, most of the SNPs were new when using significance levels of *p* = 5 × 10^−8^ and 5 × 10^−7^ (Figure 2B). The discoveries were less pronounced in the analysis of ADHD versus MGN, which revealed 19 joint variants annotated to 26 genes in the same window, 6 of which were pointed to as hits in the original GWAS (Table S9). Interestingly, 3 variants were overlapping across ADHD versus MGN and ADHD versus MCP results: rs55993747, rs79348488, and rs2027030 annotated to *KATNA1*, *ABHD17C*, and *KIF3B* genes, respectively. Enrichment for the conjFDR results also yielded >200 terms. For ADHD and MGN, they revolved around DNA and recombinational repair as well as structure resolution (e.g., “DNA double-strand break repair”) (Table S10). As for ADHD and MCP, only “nervous system development” was significant (Table S11).

We subsequently examined whether any genes or pathways identified by LAVA and conjFDR intersected with the pharmacokinetics and pharmacodynamics of paracetamol. When we compared the enrichment results for the loci retrieved in this study with known genes or the metabolization and action routes of paracetamol (Table S12), no overlap was observed.

However, DSEA revealed 3 pathways enriched for both paracetamol and genes in loci 286 and 1505 from LAVA, specifically, for locus 286, the pathways “neuron projection morphogenesis” and “cell morphogenesis involved in neuron differentiation.” Although “NCAM1 interactions” was associated with locus 1505, its *q* value did not reach the corrected significance threshold. Notably, paracetamol is ranked as the ninth drug that most upregulates this pathway of the 1308 drugs in the database. Among the results for conjFDR, 7 were overlapping with the drug-set enrichment for paracetamol, among which 1 shared locus 286, “neuron projection morphogenesis,” with LAVA (Tables S13 and S14).

### Results From the Genetic Instrumental Variable Analysis

MR methods can distinguish between vertical pleiotropy (causal pathways) and horizontal pleiotropy (noncausal association) between genetically correlated phenotypes (62). In summarized data from 5-fold larger sample sizes, we confirmed previous findings (15) demonstrating that MCP has a causal effect on ADHD and vice versa. No associations were found between ADHD and MGN given our strict criterion of significance in at least 4 models (Table S15). Heterogeneity tests and funnel plots showed overdispersion of instrumental variables, which can be translated as a sign of pleiotropy. No impact of horizontal pleiotropy or reverse causation was captured by Egger intercept and Steiger directionality tests, respectively. Leave-one-out analysis did not indicate any variant carrying the results (Table S16).

### Findings in an Independent Sample

In an independent and well-characterized clinical sample of patients with ADHD and populational control participants, we explored the robustness of PRSs for MGN and MCP in predicting clinical outcomes of ADHD. Corroborating the analysis in large-scale datasets, effects were evident only for MCP. The PRS for MCP was associated with comorbidity burden, particularly with substance use (most strongly with nicotine use disorder, *p* = .0037, odds ratio = 1.303) and bipolar disorder (*p* = .0005, odds ratio = 1.503) (Table S17).

Although we did not find an association between the MCP PRS and categorical ADHD diagnosis (Table S17), this PRS was associated with the severity of ADHD in a dimensional analysis (Table S18). The MCP PRS was correlated with increased hyperactivity/impulsivity in the total sample (*p* = .0240, *B* = 0.070), hyperactivity alone (*p* = .0128, *B* = 0.081), and total ADHD symptoms (*p* = .0053, *B* = 0.0101). When we restricted the analysis to the European subgroup, significant associations were found with inattention (*p* = .0185, *B* = 0.063) and total ADHD symptoms (*p* = .0174, *B* = 0.070).

**Figure 3:**
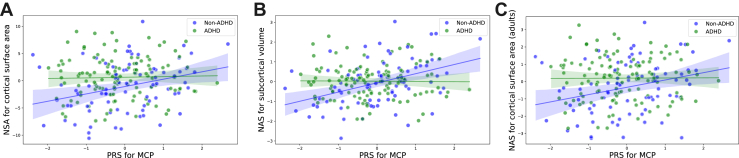
Scatter plots displaying the relationship between PRSs for MCP and brain scores for ADHD. The figure presents only brain scores with significant findings (see Table S19 for full statistics). Linear regression identified significant associations for 3 outcomes: **(A)** brain scores for cortical surface area, **(B)** subcortical volume, and **(C)** cortical surface area in adults only. Blue dots represent individuals without ADHD, while green dots represent individuals with ADHD. ADHD, attention-deficit/hyperactivity disorder; MCP, multisite chronic pain; NAS, neuroimaging association score; PRS, polygenic risk score.

Finally, significant interactions were observed between the MCP PRS and ADHD on brain scores for cortical surface area (*p* = .0349), subcortical volume (*p* = .0004), and adult cortical surface area (*p* = .0372). All associations followed the same pattern—higher MCP PRS values in control participants made their brains more similar to those of patients with ADHD (Figure 3 and Table S18).

## Discussion

We utilized various post-GWAS approaches to investigate and dissect the biological connection between ADHD, MGN, and MCP. Our findings suggest that the association between MCP and ADHD may stem from shared neurodevelopmental mechanisms underlying their significant genetic correlation. Additionally, our joint analysis of these phenotypes revealed novel genetic variants, also enriched in genes involved in nervous system development. However, further functional studies are necessary to clarify the downstream biological effects of these genes and pathways. These results also hold potential clinical significance. The strong global genetic correlations underscore the importance of further exploring the clinical relevance of the ADHD/MCP connection. Notably, MCP shares a similar level of genetic correlation with ADHD as it does with depression, anxiety, and other comorbid neuropsychiatric conditions.

MCP has been linked to genes involved in neurogenesis, synaptic plasticity, cell-cycle progression, and nervous system development (13). In contrast, MGN is a neurological condition where the activation of trigeminovascular pain pathways is believed to contribute to headache through the release of neuropeptides (63). Despite both being pain-related phenotypes, the genetic correlation between MGN and MCP was lower than that between ADHD and MCP. The involvement of the immune system in ADHD (64) and chronic pain (65) suggests that neuroinflammatory mechanisms may play a common role in the pathophysiology of both ADHD and chronic pain (6,7,66). However, our data, combined with previous findings (6), suggest that MCP could also be considered both a mental and somatic condition with a significant neurodevelopmental component. This perspective can be supported by the strong biological overlap with ADHD—a classic neurodevelopmental disorder (1,2,67)—and by the MR findings, where models from MCP to ADHD showed effect sizes ranging from 16 (IVW) to 102 (MR-Egger) times larger than those from ADHD to MCP. Nevertheless, this latter finding needs to be confirmed with a larger ADHD GWAS, as the current MCP GWAS may have more power.

Caution is advised in a consensus statement regarding the use of paracetamol during pregnancy (68). This drug is a widely used analgesic and antipyretic known for its safety, including in risk groups where nonsteroidal anti-inflammatory drugs are contraindicated, such as individuals with asthma, children, pregnant women, and nursing mothers (69). This is particularly significant, as an increasing body of epidemiological research in recent years has suggested that paracetamol use during pregnancy may be a risk factor for ADHD (70, 71, 72). The data presented here further support the notion that shared heritable factors may account for a significant portion of this association, particularly the influence of pain-related traits that might lead an individual to use such a drug. Hence, their relation to ADHD, might partially drive this observed connection (33). However, it is possible that paracetamol might still act as an environmental risk factor. The DSEA showed it can modulate the expression of genes involved in neurodevelopment, including neural morphogenesis. This aligns with findings from preclinical studies indicating that paracetamol, acting as an endocrine disrupter, impacts neurodevelopmental processes (68). However, family medicine doctors and obstetricians should consider that the benefits potentially outweigh the risks, as the effect size of the association between paracetamol use during pregnancy and the risk of developing ADHD in offspring was shown to be small (meta-analysis risk ratio = 1.25) (32). Our study paves the way for further functional investigations into the precise nature of these mechanisms and additional clinical studies aimed at distinguishing association from causation.

It is also important to mention potential pharmacological implications. There is a lack of robust information on how drugs used to treat pain can modulate ADHD symptomatology and vice versa. Two case reports describe individuals with severe chronic pain unresponsive to standard treatments. After being diagnosed with ADHD and prescribed methylphenidate, their pain improved significantly (73,74). Earlier research indicated that adults with ADHD are more sensitive to pain than control participants and that methylphenidate may have antinociceptive effects (75). These effects are further supported by recent findings showing that children with ADHD also have increased pain sensitivity, and methylphenidate helps normalize their pain experiences by raising pain thresholds (76). Additionally, a preclinical study has shown that methylphenidate can suppress mechanical and cold hyperalgesia/allodynia (77). Given these insights, clinical trials are needed to explore and confirm the potential effects of ADHD medications on pain. Moreover, using GWAS data for drug repurposing studies and identifying shared biological factors could help in establishing new pharmacological treatments.

The PRS for MCP did not show a significant association with categorical ADHD diagnosis in our study, likely due to power limitations; However, it was significantly associated with ADHD symptom severity in a dimensional analysis. Specifically, higher MCP PRS values correlated with increased hyperactivity/impulsivity and total ADHD symptoms across the entire sample, with some effects restricted to the European subgroup (e.g., inattention). These findings suggest that MCP may influence the severity of ADHD symptoms, even in the absence of a direct diagnostic classification, supporting the dimensional nature of ADHD (1). Furthermore, the observed associations between MCP PRSs and comorbid conditions, particularly substance use and bipolar disorder, highlight the broader psychiatric relevance of MCP in ADHD. The significant associations between MCP PRSs and brain structure, including cortical surface area and subcortical volume, suggest that genetic risk for MCP may influence brain development in a manner similar to that seen in ADHD. Regarding the accuracy of PRSs derived from European samples, our analysis in a Brazilian sample, particularly from the southern region where the European ancestral component is most prevalent (around 80%) (78), detected significant effects, likely due to the reduced proportion of other ancestries (African and Native American). Therefore, our sample allowed for the identification of relevant associations, highlighting the robustness of MCP PRSs even in relatively genetically diverse populations.

Our study has some limitations. Firstly, most analyses were conducted in silico following GWAS, which represented a specific aspect of a trait. While genomic data analyzed using such statistical methods are highly valuable for integrating diverse datasets (e.g., genomics, transcriptomics), functional assays are needed to better elucidate the impact of these findings. Next, pain phenotypes can vary in presentation and underlying architecture based on age and sex. For example, chronic pain prevalence is higher in adolescents, particularly in women with ADHD (79). Therefore, future analyses in larger datasets stratified by age (childhood vs. persistent ADHD) and sex could provide new insights. Additionally, our results reflect the current GWAS for the evaluated phenotypes. As the landscape broadens with larger sample sizes, so does the power to detect shared underlying biological mechanisms. This is especially important as we show that third traits can mediate these associations. For example, with increased power, other comorbid conditions should be studied using partial correlation and multivariable causal analyses. Lastly, while the sample size for our independent analysis may be modest, its detailed characterization makes it well suited for the in-depth inquiry presented here.

### Conclusions

Our study underscores the complex interplay between ADHD, MGN, and MCP, revealing shared genetic regions and variants that may drive the biological connections between ADHD and these pain-related traits. This comprehensive analysis highlights the importance of considering MCP as a relevant comorbidity in patients with ADHD, given their strong genetic overlap and shared neurodevelopmental genes, suggesting that this trait may have a partially neurodevelopmental etiology. This perspective reframes both the biological and clinical implications, opening new avenues for exploring drug effects and their consequences on brain development, and emphasizes the need for further functional investigation.

## Supplementary Material

Tables S1–S19

Key Resources Table
