# Supplementary material for: Genetic Interplay Between Attention-Deficit/Hyperactivity Disorder and Pain Suggests Neurodevelopmental Pathways and Comorbidity Risk
Source: Biol Psychiatry Glob Open Sci. 2025 Apr 25;5(4):100517. doi: 10.1016/j.bpsgos.2025.100517 (PMC12179596; doi:10.1016/j.bpsgos.2025.100517)
Supplement: Supplemental Methods and Figures S1–S3 [file mmc1.pdf]

SUPPLEMENTARY INFORMATION

Genetic Interplay Between Attention-Deficit/Hyperactivity Disorder and Pain Suggests Neurodevelopmental Pathways and Comorbidity Risk

Ciochetti *et al.*

File inventory

**Extended Methods**	01
2.1. GWAS Summary Statistics	01
2.2. Quantification of polygenic overlap	02
2.3. Local genetic correlations	02
2.4. Conjunctional FDR (conjFDR)	04
2.5. Enrichment analysis	04
2.5.1. Drug-Set Enrichment Analysis (DSEA)	05
2.6. MR: Genetic instrumental variable analysis	06
2.7. Analysis in an independent single-site case-control sample	07
2.7.1 Sample description	07
2.7.4. Neuroimaging - Brain scores calculation	08
**Supplementary Figure 1**	10
**Supplementary Figure 2**	11
**Supplementary Figure 3**	12
**References**	13

Extended Methods

2.1. GWAS Summary Statistics

The most recent genome-wide association study (GWAS) meta-analysis of Attention-Deficit/ Hyperactivity Disorder (ADHD) combined data from iPSYCH (The Lundbeck Foundation Initiative for Integrative Psychiatric Research), deCODE genetics (deCODE Genetics Inc.), and the Psychiatric Genomics Consortium (PGC), totaling 38,691 individuals with ADHD and 186,843 non-ADHD controls (1). Similarly, the GWAS meta-analysis of migraines (MGN) included 5 different samples: HUNT (Nord-Trøndelag Health Study), GeneRISK, UK Biobank, 23andMe (23andMe, Inc.) and the International Headache Genetics Consortium (IHGC2016). We acquired the available data excluding 23andMe, leading to a total of 589,356 individuals (48,975 cases and 540,381 controls) (2).

For multisite chronic pain (MCP) we utilized a GWAS composed of 412,985 individuals from the UK Biobank collection (Pan-UKB; https://pan.ukbb.broadinstitute.org; phenotype code "MCP"). This quantitative broad pain-related phenotype was defined as the sum of body parts (ranging from 0 to 7), considering head, face, neck/shoulder, back, stomach/abdomen, hip, knee, in which pain was felt for at least 3 months (3). For Major Depressive Disorder (MDD), we relied on summary statistics derived from a large-scale study that incorporated data from the Million Veteran Program, UK Biobank, and FinnGen, excluding 23andMe. This meta-analysis encompassed a cohort of 846,913 individuals, comprising 264,984 cases and 581,929 controls (4). Regarding educational attainment (EA), we utilized the most up-to-date GWAS summary statistics, which included data on years of schooling from 766,345 adult individuals, excluding the sample from 23andMe (5).

All studies utilized individuals from European ancestry only. These summary statistics underwent quality control checks. We removed single nucleotide variants (SNVs) with minor allele frequency (MAF) $\leq$ 1%, duplicated and ambiguous SNVs as well as variants in sexual chromosomes.

2.2. Quantification of total polygenic overlap

The genetic correlations between the investigated traits were computed utilizing the cross-trait Linkage Disequilibrium (LD) Score regression method (LDSC, https://github.com/bulik/ldsc) (6,7). The LSDC script first harmonizes summary statistics to contain about one million SNPs from the HapMap project, excluding the major histocompatibility complex (MHC) region. It is based on the calculation of a LD score that estimates the degree to which variants are associated with one another. It performs a regression of the SNP effect on the GWAS summary statistics of both traits on its LD score. After normalization by trait heritabilities, it yields a genetic correlation coefficient (r_g), the degree of shared variation in the genome underlying the pair of phenotypes analyzed.

Univariate and Bivariate Causal Mixture (MiXer v1.3) was also employed to quantify polygenic overlap—i.e., the proportion of variants causally associated with two traits relative to the total number of causal variants across both traits (https://github.com/precimed/mixer) (8). MiXer uses a Bayesian framework to estimate shared genetic variants regardless of effect direction. First, it utilizes a univariate mixture model to estimate the number of SNPs associated with a trait beyond LD based on GWAS association values. Next, it applies a bivariate mixture model to quantify an added effect of four instances: i) causal SNPs for trait 1 but not trait 2; ii) causal SNPs for trait 2 but not trait 1; iii) causal SNPs for both, and iv) SNPs not causal for either (9). Statistical model fit was evaluated based on likelihood maximization of signed test statistics, the Akaike Information Criterion (AIC) and predicted versus observed conditional quantile–quantile (Q-Q) plots. Venn diagrams display the proportion (Fig. 1B in the manuscript) of unique and shared SNPs between each trait pair analyzed.

2.3. Local genetic correlations

We employed the Local Analysis of [co]Variant Association (LAVA) method to identify specific genomic regions driving global genetic correlations, focusing on the estimation of local genetic correlations (local r_gs) between ADHD *vs* MGN and ADHD *vs* MCP (https://github.com/cadeleeuw/lava-partitioning) (10). LAVA achieves this by partitioning the

genome into semi-independent LD segments, referred to as loci. These loci are determined using an algorithm that enforces a minimum size requirement of 1000 SNPs (after filtering for a minor allele frequency higher than 0.01 among all SNPs), resulting in the creation of 2495 distinct loci.

To estimate the local genetic correlations, LAVA requires as inputs: i) summary statistics files for each trait (in the current study: ADHD, MGN, and MCP); ii) a reference genome in Plink format (.bim, .bed, and .fam) for LD estimation, in our case sourced from the 1000 Genomes Project; iii) an "info input" file containing the number of cases and controls for the samples; iv) a file detailing sample overlap, essential for bias correction, which is generated from a matrix with intercepts calculated for each pair of traits using LDSC; v) a file defining the loci, based on the 1000 Genomes Phase 3 Project with the GRCh37/hg19 build. This "loci coordinate' file comprises locus identifiers, chromosome coordinates, start and end points defining block boundaries, and a list of SNPs.

To quantify local r_gs for each pair of phenotypes and eliminate non-associated loci, LAVA initiates the analysis by testing the local joint univariate association for each phenotype pair. Loci exhibiting univariate associations with $P < 0.00002$ (Bonferroni correction = 0.05/2,495) for both phenotypes are then subjected to the bivariate local genetic correlation analysis. A local r_g was deemed statistically significant when the P-value, multiplied by the number of loci transitioning from univariate to bivariate analysis, was lower than 0.05 after applying Bonferroni corrections.

The partial local genetic correlation analysis was conducted following the initial analysis performed using LAVA. We used this method to evaluate the possible influence of a third trait (e.g., *Z*) in the local correlations found between two initial traits of interest (e.g., *X* and *Y*). Since there is a noteworthy genetic overlap among psychiatric disorders (11), it is commonplace to expect a degree of influence of another (third) phenotype in the resulting local correlations. This can be the case for MDD and EA, two traits more frequently associated with ADHD and pain related phenotypes. The partial correlation can be framed as the correlation between residual terms of two linear regression models, that is, $\rho XY|Z = \text{cor}(\varepsilon X, \varepsilon Y)$ for $X = Z\alpha X + \varepsilon X$ and $Y = Z\alpha Y + \varepsilon Y$. Therefore, it denotes the correlation between the initial variables that cannot be accounted for by the third trait (10).

For the significant LAVA loci resulting from the analysis of ADHD vs. MGN and ADHD vs. MCP, we first tested their bivariate local genetic correlation with either MDD or EA. For the ones that did show a significant result ($P < 0.05$), we further tested the partial genetic correlation, conditioning on one of the two or both. This way, we were partitioning out the variance of either MDD or EA to test if the original correlation between ADHD and the pain traits stood. Despite the correlations between ADHD and both MDD and EA being high (11), they are not singularities; thus, when removing that shared component, there is still some independent variance intrinsic to ADHD to be explored. This strategy was used to assess whether the significant loci are sensitive to the inclusion of relevant third phenotypes and to identify those most specific to ADHD.

2.4. Conjunctional FDR (conjFDR)

The Conjunctional False Discovery Rate (conjFDR) methodology offers significant advantages in mining comorbid genes by accurately identifying specific shared loci that surpass the significance threshold. It is a model-free approach that extends the conditional False Discovery Rate (condFDR) method (12,13) (https://github.com/precimed/pleiofdr). Under a Bayesian framework, condFDR proposes that SNPs that have shown effect in pleiotropic phenotypes have a higher probability of being truly implicated SNPs. It assesses the cumulative distribution function of nominal P-values and re-adjusts them for each SNP. The result is the SNP posterior probability of the association being null, given the observed P-value. Therefore, conjFDR is a cross-trait application of this, used to enrich for jointly associated SNPs between two phenotypes. The conjFDR value is the maximum between two condFDR values; in the case of this study, between ADHD and MGN or MCP.

2.5. Enrichment analysis

We used the start and stop base-pair coordinates to compile the genes within each segment identified by LAVA, specifically those significant after Bonferroni correction and partial correlations, using *Genome Data Viewer* (https://www.ncbi.nlm.nih.gov/genome/gdv/). Each gene-set representative of each LAVA locus was independently submitted to gene-set analysis on Enricher

(14). The databases selected were Reactome 2022 pathways, Gene Ontology Biological Process (GO BP), Cellular Component (GO CC), and Molecular Function (GO MF) (https://maayanlab.cloud/Enrichr/).

Enricher was also utilized for the enrichment of conjFDR results. Before conducting the enrichment analysis, we mapped SNPs to genes. We used ANNOVAR (ANNOtate VARiation) (15) to generate gene-based annotations (https://github.com/WGLab/doc-ANNOVAR) from the list of SNPs significant in conjFDR. For intergenic variants, the nearest upstream and downstream genes were identified. The resulting conjFDR gene-sets were then subjected to Enricher.

2.5.1 Drug-Set Enrichment Analysis (DSEA)

We obtained information on the known pharmacokinetics and pharmacodynamics pathways of paracetamol using three public domain databases: DGIdb 5.0 (16) (Drug-Gene Interaction database, https://www.dgidb.org), BindingDB (17) (https://www.bindingdb.org/rwd/bind/index.jsp), and PDSP K_i database (18) (https://pdsp.unc.edu/databases/kidb.php). Our search terms included both "paracetamol" and "acetaminophen," enabling us to gather comprehensive information on this drug.

Additionally, DSEA was used to explore other potential modes of action of paracetamol (https://dsea.tigem.it/index.php). This platform was developed to investigate shared mechanisms of different drugs that have similar or the same effects (19). We searched for the term “paracetamol” available in the platform and selected the same databases chosen in enrichment analysis (Reactome 2022, GO BP, GO CC, and GO MF). The resulting biological pathways of DSEA were crossed with the results from the gene-set analysis applied to the loci remaining significant in the LAVA approach, as well as the enrichment of the conjFDR results. In short, we looked for commonalities between the potential effects of paracetamol and the biological pathways revealed by LAVA and conjFDR.

2.6. MR: Genetic instrumental variable analysis

We performed a bidirectional two sample Mendelian Randomization (MR) between ADHD and MGN and MCP, using five different MR approaches: MR-Egger, Weighted median, Inverse Variance Weighted (IVW), Robust Adjustment Profile Score (MR-RAPS) and contamination mixture

(CONMIX). MR-Egger relaxes the assumption of 'no horizontal pleiotropy' and returns an unbiased causal effect even if this assumption is violated for all SNPs but assumes that the horizontal pleiotropic effects are not correlated with the SNP-exposure effects (inSIDE assumption) (20,21). The Weighted median approach takes the median effect of all SNPs (20) and only half the SNPs need to be valid instruments, as this method allows stronger SNPs to contribute more towards the estimate.

The Random effects IVW method performs a meta-analysis of each Wald ratio (ratio estimate for the causal effect of a biomarker on the disease), treating each SNP as a valid natural experiment. Random effects relax the assumption of horizontal pleiotropy, returning an unbiased estimate if the horizontal pleiotropy is balanced (22). MR-RAPS minimizes weak instrument bias by attributing different weights to the instrumental variables according to their associative strength (23). Finally, CONMIX identifies groups of genetic variants with similar causal estimates that may signify distinct mechanisms by which the risk factor influences the outcome. This method performs well even in the presence of invalid instrumental variables, and violation of the inSIDE assumption (24).

The implementation of these MR methods was followed by a comprehensive set of sensitivity analysis. Heterogeneity in causal effects amongst instruments is an indicator of potential violations of the MR assumptions (25). Thus, we calculated Cochran's Q heterogeneity tests. They can be calculated for the IVW, and Egger estimates. We also assessed the MR Egger regression's intercept as it represents the average pleiotropic effects across the genetic variants. Therefore, if the intercept differs from zero, we can assume directional pleiotropy (21). We used the Steiger directionality test to compute the amount of variance each SNP explains in the exposure and the outcome variable. In case of a true causal effect of the exposure on the outcome, an SNP used as an instrument should be more predictive of the exposure than the outcome, if not, it might imply reverse causation. We also generated funnel plots. Asymmetry in the funnel plot may suggest a deviation from the assumption that the genetic instrument solely influences the outcome and does not impact it through alternative pathways apart from the exposure. Finally, we carried out leave-one-out analysis to evaluate if the

MR estimate is driven or biased by a single SNP, we were able to re-estimate the effect by sequentially dropping one SNP at a time. This method informs about the sensitivity of the estimate to outliers (22).

2.7. Analysis in an independent single-site case-control sample

2.7.1 Sample description

The sample included 665 Brazilians diagnosed with ADHD from the adult division of the ADHD Outpatient Program (ProDAH-A) at Hospital de Clínicas de Porto Alegre (HCPA) and 995 blood donor controls from the same hospital. Patients self-referred to ProDAH-A following the study's public announcement in local media. All participants were 18 years or older. The mean age was 31.3 years for ADHD cases and 33.9 years for controls. The majority (97%) self-identified as White Brazilians, with no significant differences between cases (96%) and controls (97%), based on the skin color classification from the Brazilian Institute of Geography and Statistics (IBGE). The proportion of females was 44.7% among ADHD cases and 53.8% among controls. Exclusion criteria included clinically significant neurological conditions that could impair cognition, such as a history of head trauma, epilepsy, or dementia, as well as an estimated intelligence quotient (IQ) below 70. This sample has been extensively characterized in previous research (26,27).

In the Adult ADHD Porto Alegre cohort, the available GWAS data originate from 3 different waves of genotyping. The first sample batch was genotyped using PsychChip (Infinium PsychArray BeadChip; Illumina) and the remaining with Infinium Global Screening Array (GSA) BeadChip versions 1.0+MD and 3.0+MD. Pre imputation quality control (QC) and principal component analysis (PCA) were implemented in the PsychChip dataset separately and in the merged GSA batches using the Rapid Imputation and COmputational PIpeLIne (RICOPILI) for GWAS pipeline (28).

TOPMed Imputation Server was used for imputation, using Minimac4 and the TOPMed reference panel (https://imputation.biodatacatalyst.nhlbi.nih.gov/#!) (29). We selected the TOPMed reference panel for imputation due to its greater diversity compared to other panels, such as 1000 Genomes or HRC. TOPMed comprises around 100,000 samples from diverse ancestries, with

significant representation of African, European, and Native American haplotypes. Notably, approximately 15% of the reference samples are from Latino individuals, making it particularly well-suited for imputing Latin American populations. The allele frequency correlation (R^2) between our uploaded Latin American samples and the TOPMed reference panel was greater than 0.96. This strong correlation demonstrates a high degree of concordance between observed and imputed allele frequencies, underscoring the accuracy of the imputation process and the representativeness of the TOPMed reference panel for our study population. From the raw imputed dataset, more than 80% of the imputed variants had an INFO score greater than 0.8. This highlights the robustness of the imputation process, as most of the variants achieved the standard threshold for high-confidence genotype calls, ensuring the reliability of the dataset for downstream analyses.

The resulting imputed datasets underwent additional QC procedures using the following cut-offs: info < 0.8, individual and SNP call rate < 95%, MAF < 1%, HWE < 1e-06. The resulting datasets contained 419 ADHD cases, 463 controls, and 6,599,235 SNPs for PsychChip and 256 cases, 571 controls, and 7,213,318 SNPs for the merged GSA batches. These two datasets were then combined using Plink2 software, ensuring to retain only overlapping SNPs with the same chromosomal location and the same alleles referencing the same DNA strand. Additional QC, PCA, and batch analysis steps were applied to the complete dataset, resulting in 665 ADHD cases, 995 controls, and 5,751,409 SNPs. We used GWASinspector for final quality verification (https://gwasinspector.com/) (30).

2.7.4. Neuroimaging - Brain scores calculation

Brain scores are calculated similarly to polygenic risk scores (PRS); however, instead of summing the effects of each SNP, the effects of each brain region or measure are summed (31). As described in Section 2.7.4 of the Methods, different types of scores were computed, each based on a specific modality: cortical thickness, cortical surface area, and subcortical volumes. These modalities reflect the variables included in the respective scores—thickness, area, and volume. For each modality, all regions analyzed for ADHD in the latest ENIGMA (Enhancing Neuro Imaging Genetics

Through Meta Analysis) studies were considered (32,33). For instance, the subcortical volume scores included measures of regions such as the amygdala, accumbens, caudate, hippocampus, and putamen.

Each score was generated using statistics derived from the ENIGMA meta-analyses, specifically Cohen's D effect sizes and p-values for each region. This approach is analogous to the PRS calculation, which uses beta coefficients and p-values for SNPs. For a P-threshold of 1, all regions were included in the scores regardless of their association with ADHD. By contrast, a P-threshold of 0.05 included only regions significantly associated with ADHD ($p \leq 0.05$ in ENIGMA meta-analyses). For example, the cortical thickness score with a P-threshold of 0.05 included only the entorhinal, fusiform, parahippocampal, precentral, and temporal pole regions. At a threshold of 1, all 34 regions evaluated in the meta-analysis (as described in reference number(34)) were included.

After filtering brain regions based on their p-values of association with ADHD, Cohen's D effect sizes for each region were used as weights in the calculation, analogous to beta coefficients for SNPs in PRS. Like PRSs, brain scores lack a specific unit. The variables in our dataset are measured in millimeters (cortical thickness), square millimeters (cortical surface area), and cubic millimeters (subcortical volumes). Therefore, we used standardized Z-values in the linear regression analysis. Finally, internal consistency was assessed using Cronbach's alpha, with a threshold of 0.7 considered acceptable, to evaluate how well the set of measures reflects a single latent construct. This analysis considered: (1) **Item covariances**: Cronbach's alpha is based on the average pairwise covariances (or correlations) between items. Higher covariances indicate greater consistency in measuring the same construct, leading to a higher alpha value; (2) **Number of items**: A greater number of items generally increases alpha, provided they measure the same underlying construct; (3) **Variance of the total scale**: The overall variance of the scale (sum of all item scores) contributes to alpha. Higher total variance relative to individual item variances results in a higher alpha; (4) **Item variance**: Low variance among items can reduce alpha, as it limits the ability to discriminate among respondents; and (5) **Item homogeneity**: High homogeneity indicates that the items measure the same construct. Conversely, heterogeneity reduces alpha if items measure multiple constructs.

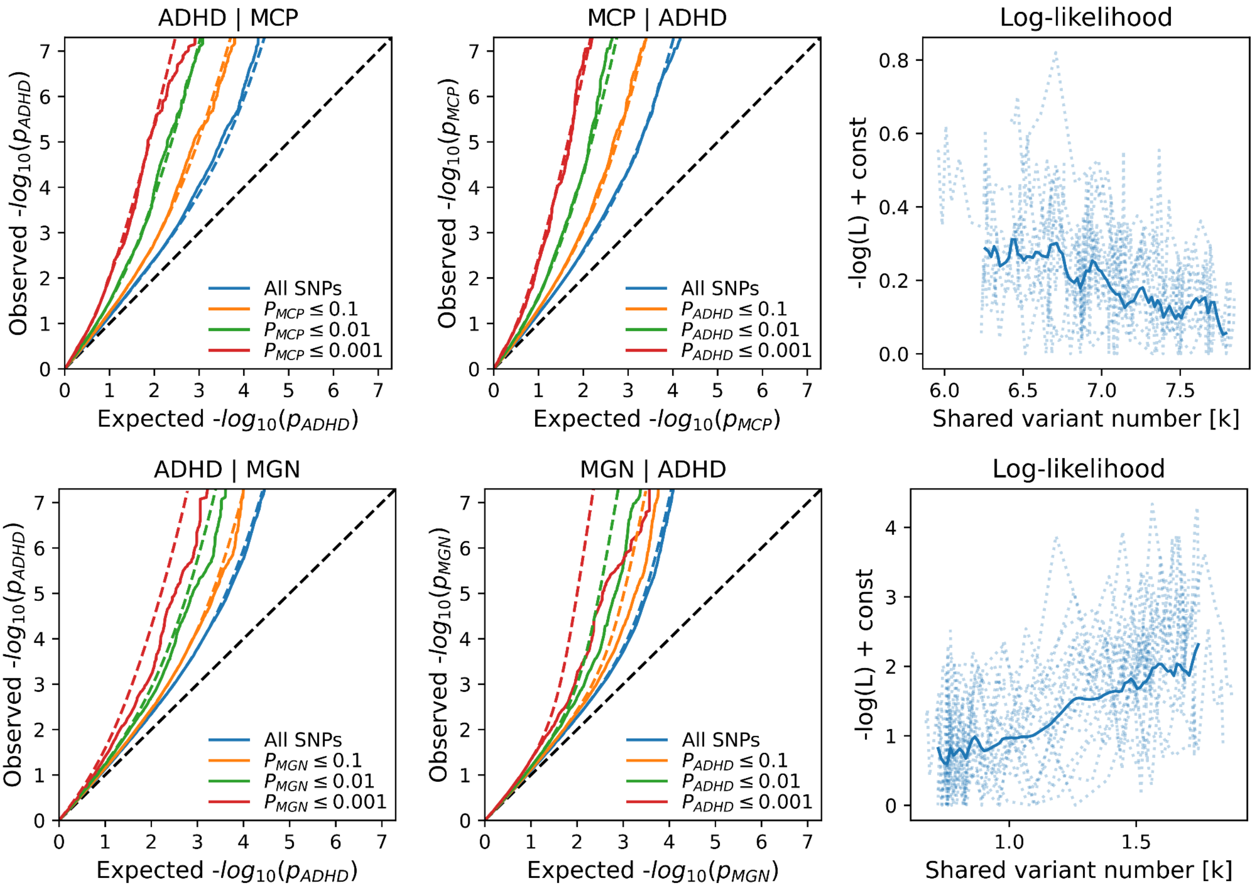

Supplementary Figure 1 - Complementary MiXer plots. Conditional Quantile-Quantile plots of observed *versus* expected -log10(p) of P values in phenotype 1 as a function of different thresholds of association with phenotype 2. The plots show the distribution according to the null hypothesis of no association (black line), at P ≤ 0.001 (red), P ≤ 0.01 (green), P ≤ 0.1 (blue) and all single nucleotide polymorphisms (SNPs). The dotted lines refer to MiXer predictions. Bivariate analysis took into account the linkage disequilibrium (LD) structure, a random pruning at cut-offs of minor allele frequency (MAF) = 0.05, r^2 = 0.8 and 20 iterations (a set of ~600K SNPs per run). Lastly, a log-likelihood graph as a function of polygenic overlap shows the balance of model-cost *versus* polygenic overlap: the minimal model is the one furthest to the right, the maximum model is furthest to the left and the best model is the lowest point. MiXer employs Akaike and Bayesian Information Criterion (AIC and BIC) to evaluate the best versus other models. The summary statistics were not constrained to HapMap variants and were formatted for MiXer independently from the LDSC formatting process.
ADHD: Attention deficit hyperactivity disorder; MCP: Multisite chronic pain; MGN: migraines.

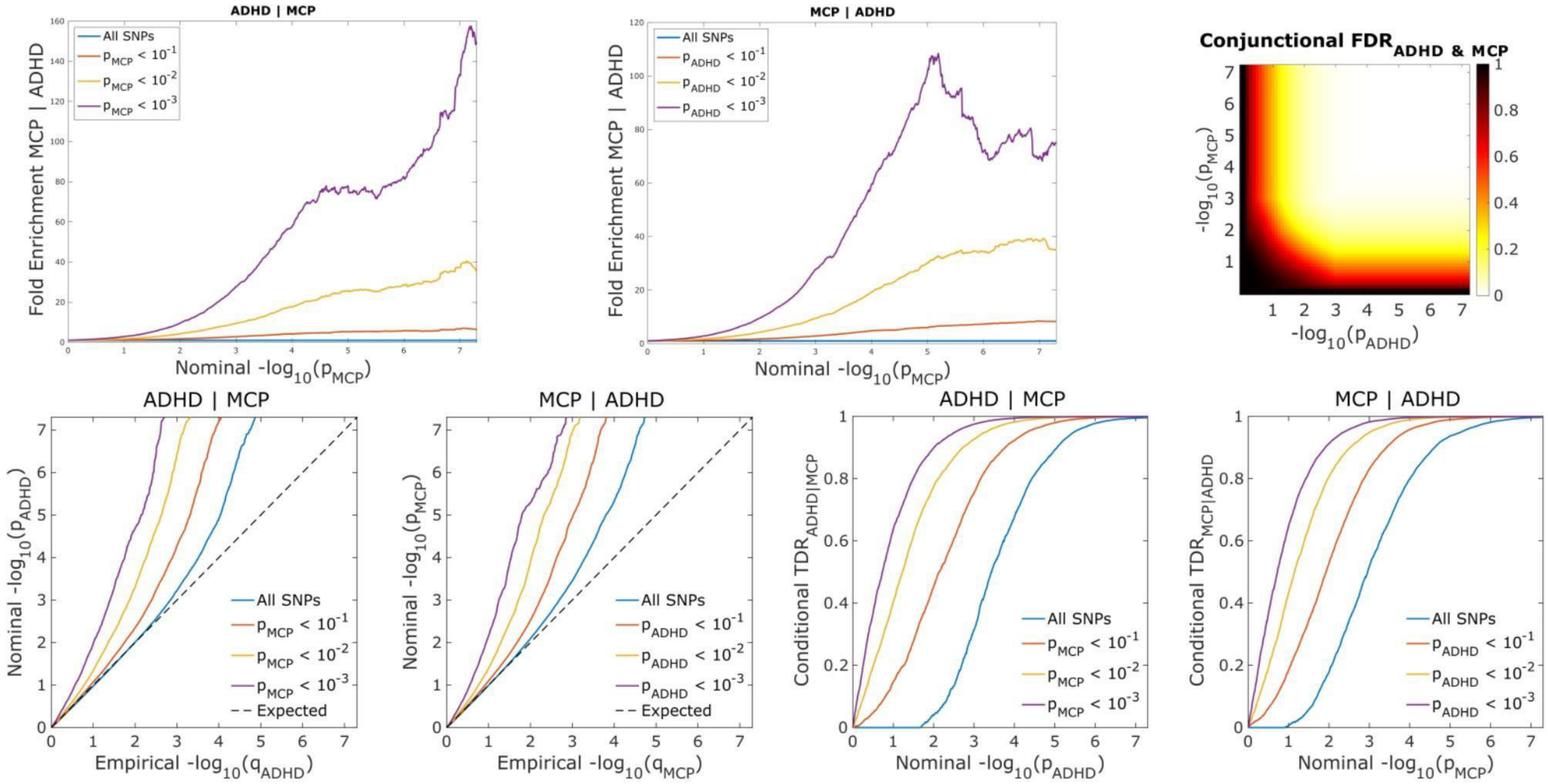

Supplementary Figure 2 - Full Conjunctional False Discovery Rate (conjFDR) Plot for ADHD vs multisite chronic pain (MCP). Results of the conjFDR analysis for ADHD vs MCP. The Quantile-Quantile (Q-Q) plots demonstrate cross-trait enrichment between these phenotypes. We used 500 iterations of "randprune_n", as recommended by the conjFDR developers, instead of the default of 20, with an LD threshold of $r^2=0.1$. We excluded the major histocompatibility complex (MHC) and the 8p21 region from the analysis to avoid biases due to complex LD patterns. The conjFDR 2D look-up table displays the FDR values corresponding to these cross-trait enrichments, with the color scheme indicating FDR values.

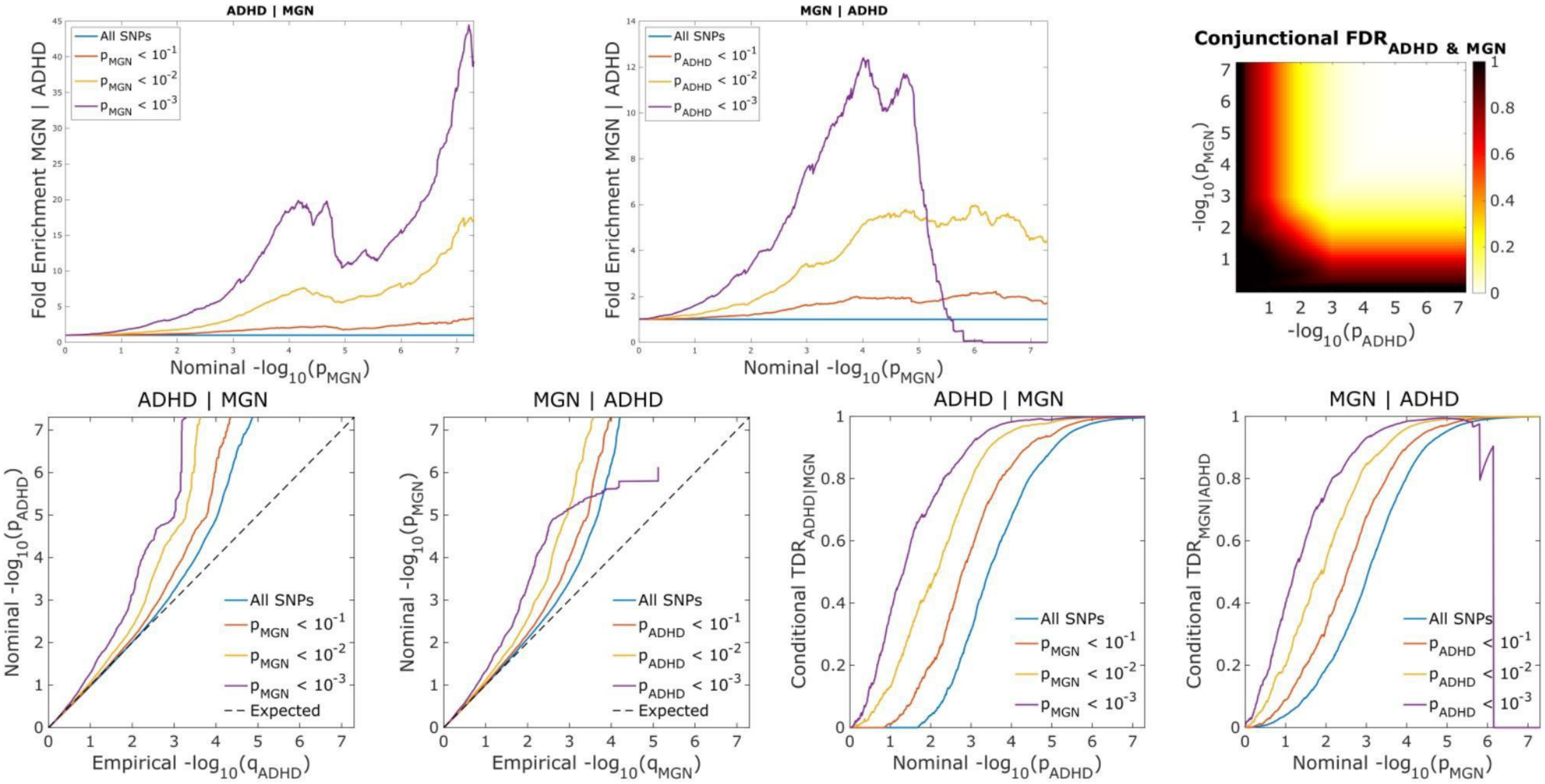

Supplementary Figure 3 - Full Conjunctional False Discovery Rate (conjFDR) Plot for ADHD vs migraines (MGN). Results of the conjFDR analysis for ADHD vs MGN. The Quantile-Quantile (Q-Q) plots demonstrate cross-trait enrichment between these phenotypes. We used 500 iterations of "randprune_n", as recommended by the conjFDR developers, instead of the default of 20, with an LD threshold of r^2=0.1. We excluded the major histocompatibility complex (MHC) and the 8p21 region from the analysis to avoid biases due to complex LD patterns. The conjFDR 2D look-up table displays the FDR values corresponding to these cross-trait enrichments, with the color scheme indicating FDR values.

References

1. Demontis D, Walters GB, Athanasiadis G, Walters R, Therrien K, Nielsen TT, *et al.* (2023): Genome-wide analyses of ADHD identify 27 risk loci, refine the genetic architecture and implicate several cognitive domains. *Nat Genet* 55: 198–208.

2. Hautakangas H, Winsvold BS, Ruotsalainen SE, Bjornsdottir G, Harder AVE, Kogelman LJA, *et al.* (2022): Genome-wide analysis of 102,084 migraine cases identifies 123 risk loci and subtype-specific risk alleles. *Nat Genet* 54: 152–160.

3. Johnston KJA, Huckins LM (2023): Chronic Pain and Psychiatric Conditions. *Complex Psychiatry* 9: 24–43.

4. Levey DF, Stein MB, Wendt FR, Pathak GA, Zhou H, Aslan M, *et al.* (2021): Bi-ancestral depression GWAS in the Million Veteran Program and meta-analysis in >1.2 million individuals highlight new therapeutic directions. *Nat Neurosci* 24: 954–963.

5. Okbay A, Wu Y, Wang N, Jayashankar H, Bennett M, Nehzati SM, *et al.* (2022): Polygenic prediction of educational attainment within and between families from genome-wide association analyses in 3 million individuals. *Nat Genet* 54: 437–449.

6. Bulik-Sullivan B, Loh P-R, Finucane HK, Ripke S, Yang J, Schizophrenia Working Group of the Psychiatric Genomics Consortium, *et al.* (2015): LD Score regression distinguishes confounding from polygenicity in genome-wide association studies. *Nat Genet* 47: 291–295.

7. Bulik-Sullivan B, Finucane HK, Anttila V, Gusev A, Day FR, Loh P-R, *et al.* (2015): An atlas of genetic correlations across human diseases and traits. *Nat Genet* 47: 1236–1241.

8. Frei O, Holland D, Smeland OB, Shadrin AA, Fan CC, Maeland S, *et al.* (2019): Bivariate causal mixture model quantifies polygenic overlap between complex traits beyond genetic correlation. *Nat Commun* 10: 2417.

9. Bahrami S, Hindley G, Winsvold BS, O'Connell KS, Frei O, Shadrin A, *et al.* (2022): Dissecting the shared genetic basis of migraine and mental disorders using novel statistical tools. *Brain*

J Neurol 145: 142–153.

10. Werme J, van der Sluis S, Posthuma D, de Leeuw CA (2022): An integrated framework for local genetic correlation analysis. *Nat Genet* 54: 274–282.
11. The Brainstorm Consortium, Anttila V, Bulik-Sullivan B, Finucane HK, Walters RK, Bras J, *et al.* (2018): Analysis of shared heritability in common disorders of the brain. *Science* 360: eaap8757.
12. Smeland OB, Frei O, Shadrin A, O'Connell K, Fan C-C, Bahrami S, *et al.* (2020): Discovery of shared genomic loci using the conditional false discovery rate approach. *Hum Genet* 139: 85–94.
13. Andreassen OA, Thompson WK, Schork AJ, Ripke S, Mattingsdal M, Kelsoe JR, *et al.* (2013): Improved detection of common variants associated with schizophrenia and bipolar disorder using pleiotropy-informed conditional false discovery rate. *PLoS Genet* 9: e1003455.
14. Xie Z, Bailey A, Kuleshov MV, Clarke DJB, Evangelista JE, Jenkins SL, *et al.* (2021): Gene Set Knowledge Discovery with Enrichr. *Curr Protoc* 1: e90.
15. Wang K, Li M, Hakonarson H (2010): ANNOVAR: functional annotation of genetic variants from high-throughput sequencing data. *Nucleic Acids Res* 38: e164.
16. Cannon M, Stevenson J, Stahl K, Basu R, Coffman A, Kiwala S, *et al.* (2024): DGIdb 5.0: rebuilding the drug-gene interaction database for precision medicine and drug discovery platforms. *Nucleic Acids Res* 52: D1227–D1235.
17. Liu T, Lin Y, Wen X, Jorissen RN, Gilson MK (2007): BindingDB: a web-accessible database of experimentally determined protein–ligand binding affinities. *Nucleic Acids Res* 35: D198–D201.
18. Roth BL, Lopez E, Patel S, Kroeze WK (2000): The Multiplicity of Serotonin Receptors: Uselessly Diverse Molecules or an Embarrassment of Riches? *The Neuroscientist* 6: 252–262.

19. Napolitano F, Sirci F, Carrella D, di Bernardo D (2016): Drug-set enrichment analysis: a novel tool to investigate drug mode of action. *Bioinformatics* 32: 235–241.

20. Bowden J, Del Greco M F, Minelli C, Davey Smith G, Sheehan NA, Thompson JR (2016): Assessing the suitability of summary data for two-sample Mendelian randomization analyses using MR-Egger regression: the role of the I2 statistic. *Int J Epidemiol* 45: 1961–1974.

21. Bowden J, Davey Smith G, Burgess S (2015): Mendelian randomization with invalid instruments: effect estimation and bias detection through Egger regression. *Int J Epidemiol* 44: 512–525.

22. Hemani G, Zheng J, Elsworth B, Wade KH, Haberland V, Baird D, *et al.* (2018): The MR-Base platform supports systematic causal inference across the human phenome ((R. Loos, editor)). *eLife* 7: e34408.

23. Zhao Q, Wang J, Hemani G, Bowden J, Small DS (2020): Statistical inference in two-sample summary-data Mendelian randomization using robust adjusted profile score. *Ann Stat* 48: 1742–1769.

24. Slob EAW, Burgess S (2020): A comparison of robust Mendelian randomization methods using summary data. *Genet Epidemiol* 44: 313–329.

25. Bowden J, Del Greco M F, Minelli C, Davey Smith G, Sheehan N, Thompson J (2017): A framework for the investigation of pleiotropy in two-sample summary data Mendelian randomization. *Stat Med* 36: 1783–1802.

26. Grevet EH, Bandeira CE, Vitola ES, de Araujo Tavares ME, Breda V, Zeni G, *et al.* (2024): The course of attention-deficit/hyperactivity disorder through midlife. *Eur Arch Psychiatry Clin Neurosci* 274: 59–70.

27. Karam RG, Breda V, Picon FA, Rovaris DL, Victor MM, Salgado C a. I, *et al.* (2015): Persistence and remission of ADHD during adulthood: a 7-year clinical follow-up study.

Psychol Med 45: 2045–2056.

28. Lam M, Awasthi S, Watson HJ, Goldstein J, Panagiotaropoulou G, Trubetskoy V, *et al.* (2020): RICOPILI: Rapid Imputation for COnsortias PIpeLIne. *Bioinforma Oxf Engl* 36: 930–933.
29. Taliun D, Harris DN, Kessler MD, Carlson J, Szpiech ZA, Torres R, *et al.* (2021): Sequencing of 53,831 diverse genomes from the NHLBI TOPMed Program. *Nature* 590: 290–299.
30. Ani A, van der Most PJ, Snieder H, Vaez A, Nolte IM (2021): GWASinspector: comprehensive quality control of genome-wide association study results. *Bioinformatics* 37: 129–130.
31. Axelrud LK, Simioni AR, Pine DS, Winkler AM, Pan PM, Sato JR, *et al.* (2021): Neuroimaging Association Scores: reliability and validity of aggregate measures of brain structural features linked to mental disorders in youth. *Eur Child Adolesc Psychiatry* 30: 1895–1906.
32. Hoogman M, Bralten J, Hibar DP, Mennes M, Zwiers MP, Schweren LSJ, *et al.* (2017): Subcortical brain volume differences in participants with attention deficit hyperactivity disorder in children and adults: a cross-sectional mega-analysis. *Lancet Psychiatry* 4: 310–319.
33. Hoogman M, Muetzel R, Guimaraes JP, Shumskaya E, Mennes M, Zwiers MP, *et al.* (2019): Brain Imaging of the Cortex in ADHD: A Coordinated Analysis of Large-Scale Clinical and Population-Based Samples. *Am J Psychiatry* 176: 531–542.
34. Bandeira CE, Grevet EH, Vitola ES, da Silva BS, Cupertino RB, Picon FA, *et al.* (2024): Exploring Neuroimaging Association Scores in adulthood ADHD and middle-age trajectories. *J Psychiatr Res* 176: 348–353.
